# Antibacterial activity of statins: a comparative study of Atorvastatin, Simvastatin, and Rosuvastatin

**DOI:** 10.1186/1476-0711-11-13

**Published:** 2012-05-07

**Authors:** Majed Masadeh, Nizar Mhaidat, Karem Alzoubi, Sayer Al-azzam, Ziad Alnasser

**Affiliations:** 1 Faculty of Pharmacy, Jordan University of Science & Technology, Irbid, 22110, Jordan; 2 Faculty of Medicine, Jordan University of Science & Technology, Irbid, 22110, Jordan

**Keywords:** Antimicrobial activity, Statins, Atorvastatin, Simvastatin, Rosuvastatin

## Abstract

**Background:**

Statins have several effects beyond their well-known antihyperlipidemic activity, which include immunomodulatory, antioxidative and anticoagulant effects. In this study, we have tested the possible antimicrobial activity of statins against a range of standard bacterial strains and bacterial clinical isolates.

**Methods:**

Minimum inhibitory concentrations (MIC) values were evaluated and compared among three members of the statins drug (atorvastatin, simvastatin, and rosuvastatin).

**Results:**

It was revealed that statins are able to induce variable degrees of antibacterial activity with atorvastatin, and simvastatin being the more potent than rosuvastatin. *Methicillin-sensitive staphylococcus aureus (MSSA), methicillin-resistant staphylococcus aureus (MRSA), vancomycin-susceptible enterococci (VSE), vancomycin-resistant enterococcus (VRE), acinetobacter baumannii, staphylococcus epidermidis*, and *enterobacter aerogenes,* were more sensitive to both atorvastatin, and simvastatin compared to rosuvastatin. On the other hand, *escherichia coli, proteus mirabilis, and enterobacter cloacae* were more sensitive to atorvastatin compared to both simvastatin and rosuvastatin. Furthermore, most clinical isolates were less sensitive to statins compared to their corresponding standard strains.

**Conclusion:**

Our findings might raise the possibility of a potentially important antibacterial class effect for statins especially, atorvastatin and simvastatin.

## Background

Statins, also known as 3-hydroxy-3-methyl-glutaryl (HMG)-CoA reductase inhibitors, are a potent antihyperlipidemic drug group that is widely used for the treatment of hyperlipidemia. The HMG-CoA reductase is the enzyme responsible for the rate-limiting step in the cholesterol synthesis mevalonate pathway [1]. HMG-CoA inhibition results in a reduction of cholesterol synthesis and an increase in the synthesis of low-density-lipoprotein receptors. This, results in increased clearance of LDL cholesterol from the blood stream [2].

HMG-CoA reductase inhibitors are known to have effects beyond their lipid lowering effects, collectively known as pleiotropic effects [3]. These pleiotropic effects result in improvement of endothelial function, modulation of inflammatory responses and antioxidant effects, maintenance of plaque stability, and prevention of thrombus formation [4-6]. The area of pleiotropic effects of statins is promising and several such effects are being speculated.

Statins have also been investigated for their antibacterial action. In one study of the role of statins in community acquired pneumonia, [7] statins were shown to have immunomodulatory, and antioxidative actions, and a significant effect on the concentrations systemic cytokine [8-12]. Several animal studies [9,13-17] and observational studies in humans [18-22] have shown that individuals treated with statins are less prone to bacterial infection and present better outcomes. The antibacterial and anti-inflammatory effects of statins were investigated in a meta-analysis, which suggested that statin use may be associated with useful outcomes in the treatment and prevention of different infections in recipients of solid-organ transplants [23,24]. This study aims to further investigate the antibacterial action of statins and identify their spectrum of action.

## Methods

### Microbial culture and growth conditions

Antibacterial activity of statins was evaluated against different reference bacteria including *E. coli* ATTC 35218, *Pseudomonas aeruginosa* ATTC 9027, *MSSA* ATTC 25213, *MRSA* ATTC 43300, *Streptococcus pneumoniae* ATTC 25923, *VSE* ATTC 19433, *VRE* ATTC 51299, *A. baumannii* ATTC 17978, *P. mirabilis* ATTC 12459, *Klebsiella pneumoniae* ATTC 13883, *Streptococcus pyogenes* ATTC 19615, *Haemophilus influenzae* ATTC 29247, *S. epidermidis* ATTC 12228, *E. aerogenes* ATTC 29751, *Citrobacter freundii* ATTC 8090, *E. cloacae* ATTC 13047, and against clinical isolates. Eighty clinical isolates were used in this study, comprising 14 different bacterial species. They were obtained from non-duplicate clinical specimens, including ear swab, throat swab, vaginal swab, sputum, urine, and blood culture, from the Microbiology Laboratory at King Abdullah University Hospital (KAUH) in north of Jordan, between April and September of 2010.

The organisms were stored at −70°C in trypticase-soy broth and 20% glycerol (BBL Microbiology Systems, Md, USA). When ready for batch susceptibility testing, samples were thawed. To ensure purity and viability, samples were, then, passed 3 times. Minimum inhibitory concentrations (MICs) were determined in accordance with the Clinical and Laboratory Standards Institute (CLSI) [25].

### Determination of minimum inhibitory concentration (MIC)

The MIC was determined by serial dilution method according to the National Committee for Clinical Laboratory Standards [26]. Briefly, statins were serially diluted, and added to plates containing molten BBL Muller-Hinton Gold II agar (BBL Microbiology Systems). Thereafter, plates were slightly cooled and dried. Then, using an a steer replicator, aliquots containing about 5 × 10^4^ colony forming units per drop of different bacterial strains were placed in each plate. After an 18-hour incubation period at 37°C, plates were read. MIC was defined as the lowest concentration at which no growth, a faint haze or fewer than 3 discrete colonies were detected. Plates were read in duplicate, and the highest MIC value was recorded. The breakpoints indicated in the tables of the National Committee for CLSI [26] were used to determine susceptibility and resistance.

### Chemicals

Simvastatin atorvastatin and Rosuvastatin were a generous gift from Advanced Pharmaceutical Industries (Amman, Jordan). Drugs (simvastatin, atorvastatin and rosuvastatin) were dissolved in DMSO to a stock concentration of 1 mg/ml, and they were used for MIC determination. All drugs were used as raw materials. DMSO was used to help in dissolving the drugs used. As DMSO is known for possessing no antibacterial activity of its own, DMSO/no statin served as a negative control.

### Statistics

Analysis was performed using GraphPad Prism software (version 4.0, GraphPad software, LA jolle, CA). One-way ANOVA followed by Tukey’s post-test were used to determine if there was any statistically significant difference. *P*-values <0.05 was considered significant.

## Results

The antibacterial activity of atorvastatin, simvastatin, and rosuvastatin were investigated against 16 standard bacterial strains. Results shown in Table 1 revealed that statins are able to induce variable degrees of antibacterial activity, where atorvastatin and simvastatin are the most potent. *MSSA, MRSA, VSE, VRE, A. baumannii, S. epidermidis*, and *E. aerogenes,* were more sensitive to both atorvastatin, and simvastatin compared to rosuvastatin (*P* < 0-05). On the other hand, *E. coli, P. mirabilis, and E. cloacae* were more sensitive to atorvastatin compared to both simvastatin and rosuvastatin (*P* < 0.05).

**Table 1:** Minimum inhibitory concentrations (MIC; μg/mL) of different statins against standard bacteria

**Statins**	**Rosuvastatin**	**Atorvastatin**	**Simvastatin**
	MIC; μg/mL	MIC; μg/mL	MIC; μg/mL
*E. coli* ATTC 35218	104.17 ± 36.08	26.04 ± 9.02*	52.08 ± 18.04
*P. aeruginosa* ATTC 9027	166.67 ± 72.16	83.33 ± 36.08	166.67 ± 72.16
*MSSA* ATTC 25213	208.33 ± 72.16	41.67 ± 18.04*	26.04 ± 9.02*
*MRSA* ATTC 43300	500 ± 0.00	83.33 ± 36.08*	166.67 ± 72.16*
*S. pneumoniae* ATTC 25923	333.33 ± 144.33	104.17 ± 36.08	166.67 ± 72.16
*VSE* ATTC 19433	333.33 ± 144.33	83.33 ± 36.08*	52.08 ± 18.04*
*VRE* ATTC 51299	500 ± 0.00	166.67 ± 72.16*	104.17 ± 36.08*
*A. baumannii* ATTC 17978	333.33 ± 144.33	15.62 ± 0.00*	104.17 ± 36.08*
*P. mirabilis* ATTC 12459	250 ± 0.00	62.5 ± 0.00*	166.67 ± 72.16
*K. pneumoniae* ATTC 13883	333.33 ± 144.33	166.67 ± 72.16	166.67 ± 72.16
*S. pyogenes* ATTC 19615	166.67 ± 72.16	83.33 ± 36.08	62.5 ± 0.00
*H. influenza*e ATTC 29247	166.67 ± 72.16	83.33 ± 36.084	52.08 ± 18.04
*S. epidermidis* ATTC 12228	166.67 ± 72.16	20.83 ± 9.02*	26.04 ± 9.02*
*E. aerogenes* ATTC 29751	104.17 ± 36.08	15.62 ± 0.00*	26.04 ± 9.02*
*C. freundii* ATTC 8090	166.67 ± 72.16	83.33 ± 36.08	52.08 ± 18.04
*E. cloacae* ATTC 13047	166.67 ± 72.16	41.67 ± 18.04*	62.5 ± 0.00

MICs were determined using serial dilution method according to the procedures National Committee for Clinical Laboratory Standards.

* indicates significant difference from rosuvastatin group.

We next studied the antibacterial activity of statins against 16 clinical isolates of bacteria by measuring MIC values. Most clinical isolates were less sensitive to statins compared to their corresponding standard strains (Table 2). In addition, when compared for their antibacterial activity atorvastatin and simvastatin were significantly more potent compared to Rosuvastatin. For example, *P. aeruginosa, MSSA, MRSA, S. pneumonia, VRE, A. baumannii, H. influenza, S. epidermidis, E. aerogenes, C. freundii,* and *E. cloacae* were more sensitive to atorvastatin and simvastatin compared to rosuvastatin (*P* < 0-05, Table2). Additionally, VSE and VRE isolates were significantly more sensitive to atorvastatin compared to simvastatin (*P* < 0-05, Table 2).

**Table 2:** Minimum inhibitory concentrations (MIC; μg/mL) of different statins against different clinical isolates

**Clinical Isolate**	**Rosuvastatin**	**Atorvastatin**	**Simvastatin**
	MIC; μg/mL	MIC; μg/mL	MIC; μg/mL
*E. coli* Isolates	125.00 ± 16.14	100.00 ± 33.75	112.5 ± 30.19
*P. aeruginosa* Isolates	291.67 ± 39.53	95.83 ± 22.09*	120.83 ± 32.27*
*MSSA* Isolates	341.67 ± 20.84	52.08 ± 11.04*	60.42 ± 12.76*
*MRSA* Isolates	500.00 ± 0.00	108.33 ± 27.36*	116.67 ± 30.19*
*S. pneumoniae* Isolates	416.67 ± 0.00	229.17 ± 60.38*	291.67 ± 39.53*
*VSE* Isolates	333.33 ± 0.00	95.83 ± 22.09*	291.67 ± 39.53#
*VRE* Isolates	500.00 ± 0.00	216.67 ± 32.27*	291.67 ± 39.53*#
*A. baumannii* Isolates	300.00 ± 79.05	21.87 ± 4.94*	32.29 ± 6.38*
*P. mirabilis* Isolates	191.67 ± 32.27	127.08 ± 25.51	158.33 ± 32.27
*K. pneumoniae* Isolates	258.33 ± 64.55	216.67 ± 51.03	241.67 ± 60.38
*S. pyogenes* Isolates	275.00 ± 72.17	133.33 ± 19.76	145.83 ± 32.27
*H. influenza*e Isolates	366.67 ± 0.00	104.17 ± 36.08*	145.83 ± 32.27*
*S. epidermidis* Isolates	233.33 ± 39.52	19.78 ± 4.94*	35.41 ± 4.94*
*E. aerogenes* Isolates	183.33 ± 0.00	19.78 ± 4.94*	33.33 ± 4.94*
*C. freundii* Isolates	333.33 ± 79.06	108.33 ± 27.36*	133.33 ± 39.58*
*E. cloacae* Isolates	316.67 ± 64.55	113.54 ± 27.06*	143.75 ± 36.97*

MICs were determined using serial dilution method according to the procedures National Committee for Clinical Laboratory Standards.

* indicates significant difference from rosuvastatin group. # indicates significant difference from atorvastatin group.

## Discussion

The emergence of drug resistance with patient’s poor compliance, drugs adverse effects and the higher cost of therapy combinations, indicates a strong need for a therapy regimens with similar or higher antibiotics beneficial properties but with better adverse effects profiles. Results of the current study suggest a class effect antibacterial activity for statins, and indicate the superiority of the antibacterial activity of atorvastatin and simvastatin against several standard bacterial strains and clinical isolates as compared to rosuvastatin.

Statins were demonstrated to have pharmacological actions beyond their antihyperlipdimic properties including immunomodulatory, antioxidative and anticoagulant effects. A recent study indicated a direct antimicrobial effect of simvastatin and to a lesser extent fluvastatin against *MSSA* and *MRSA*[27]. Another study showed the antibacterial effect of atorvastatin and rosuvastatin in Gram + and Gram– bacteria [28]. Results of the present study extend those of previous studies to include more agents of the statins family and test these agents against a wide range of standard bacterial strains and clinical isolates.

A very recent study has reported MIC values for simvastatin against *S. pneumoniae* and *M. catarrhalis* that are similar to the ones reported in this study [29]. These MIC values reflect concentrations of statins that are higher than regular concentrations detected in human blood during statins therapy [30]*.* However, since multiple dose statins are known for their favorable effect on the course of bacterial infections [18-22], it is possible that statins undergoes accumulation at target human tissues upon multiple dosing, or there could a formation of relevant breakdown products *in vivo*. Alternatively, statins could aid the action of other antibacterial agent during the treatment of infections in human through their reported pleiotropic actions [31-33].

Statins induce their antihyperlipdimic, via inhibition of HMG-CoA reductase. In bacterial cells, HMG-CoA reductase is essential, where it is required for the biosynthesis of isoprenes [34]. However, bacterial HMG-CoA reductase is of a different structural class with an affinity for statins that is 10 000 times weaker than the enzyme found in eukaryotes [34]. Thus, it is unlikely that antibacterial activity of statins can be attributed to the known mechanism of action (i.e. inhibition of HMG-CoA reductase). Other possible mechanisms could be related to the pleiotropic properties of statins. For example, multiple statins including atorvastatin and simvastatin, were shown to be cytotoxic, to suppress cells growth, and to promote apoptosis [31-33]. It is possible that the currently reported antibacterial activity of statins is related to such effects.

Results of the current study showed the superiority of the antibacterial effcets of atorvastatin or simvastatin to that of rosuvastatin. Previous studies have reported distinct effects, other than the antibacterial activity, for atorvastatin and simvastatin, compared to other members of statins [35,36]. Additionally, our results show that atrovastatin was superior to simvastatin against VSE and VRE clinical isolates. These distinct effects could also be related to the differences in chemical structure among statins. For example, simvastatin is naturally product of fungal fermentation, whereas atorvastatin is a chemically synthesized derivative. Additionally, satins differ in their lipids affinity, thus, they could have different intrinsic activities. However, these points need more study, and could be a matter of future work.

## Conclusion

In summary, results of the current study raise the possibility of a potentially important class effect and future studies are recommended to elucidate mechanism (s) by which atorvastatin and simvastatin are inducing their antibacterial effects.

## Competing interests

The authors declare that they have no competing interests.

## Authors’ contributions

MM carried out MIC determination studies, and participated in drafting the manuscript. NM participated in MIC determination studies, and study design, and helped in drafting the manuscript. KA participated in the design of the study, performed the statistical analysis, and drafted the manuscript. SA conceived of the study, and participated in its design and coordination and helped to draft the manuscript. ZA participated in the design of the study, prepared clinical isolates, and helped to draft the manuscript. All authors read and approved the final manuscript.
